# High-order alpha-band network reorganization underpins clinical symptom alleviation following EEG neurofeedback in preschool children with autism spectrum disorder: a multidimensional study

**DOI:** 10.3389/fnhum.2026.1897176

**Published:** 2026-07-14

**Authors:** Jiannan Kang, Yuqi Li, Xiaoli Li, He Chen

**Affiliations:** 1 Child Rehabilitation Division, Ningbo Rehabilitation Hospital, Ningbo, China; 2 State Key Laboratory of Cognitive Neuroscience and Learning, Beijing Normal University, Beijing, China; 3 School of Automation Science and Engineering, South China University of Technology, Guangzhou, China

**Keywords:** autism spectrum disorder, children, EEG neurofeedback, high-order functional connectivity, network state entropy

## Abstract

**Background:**

Autism Spectrum Disorder (ASD) is increasingly recognized as a disorder of neural connectivity and network architecture. While EEG neurofeedback (NFB) has shown clinical efficacy in ameliorating ASD symptoms, the underlying neurophysiological mechanisms—specifically regarding high-order network reorganization beyond simple power spectrum changes—remain poorly understood. Furthermore, there is a need to validate whether robust biomarkers can be identified using low-density EEG systems suitable for preschool children.

**Methods:**

In this randomized, sham-controlled trial, 40 preschool children with ASD (mean age 5.56 years) were assigned to either an active NFB group (*n* = 20) or a sham-control group (*n* = 20). The experimental group underwent a 4-week alpha-band NFB training protocol using an 8-channel portable EEG system. We employed a multidimensional analytical framework integrating High-Order Functional Connectivity (HOFC), dynamic Network State Entropy (NSE), and cross-frequency interaction analysis to characterize neural reorganization. Clinical improvements were assessed using the Autism Behavior Checklist (ABC).

**Results:**

The NFB group exhibited significant reductions in ABC total scores and Social Relating subscale scores. Neurophysiologically, while conventional Low-Order Functional Connectivity (LOFC) failed to capture significant global changes, the alpha-band HOFC revealed a systemic optimization toward a more efficient small-world architecture, characterized by increased Clustering Coefficient (CC) and Local Efficiency (LE), and decreased Characteristic Path Length (CPL). Additionally, NSE analysis indicated increased dynamic flexibility across multiple frequency bands, reflecting a reduction in neural rigidity. Crucially, the optimization of alpha-band HOFC metrics was significantly correlated with the degree of clinical symptom alleviation (*r* = 0.678, *p* = 0.015).

**Conclusion:**

These findings demonstrate that alpha-band NFB facilitates clinical recovery in preschool children with ASD by inducing frequency-specific high-order network reorganization. The study suggests that alpha-band HOFC may serve as a potential exploratory electrophysiological correlate for monitoring therapeutic responses.

## 1 Introduction

Autism Spectrum Disorder (ASD) is a complex neurodevelopmental condition characterized by persistent deficits in social communication and restricted (Quilty et al., 2013), repetitive patterns of behavior (Bhat, 2021). Pathophysiologically, ASD is increasingly recognized as a disorder of connectivity (Belmonte et al., 2004), marked by an imbalance between excitatory and inhibitory (E/I) neurotransmission and a disorganized neural network architecture (Rubenstein and Merzenich, 2003). These disruptions hinder the brain’s ability to integrate information across distant regions, leading to the heterogeneous clinical manifestations observed in pediatric populations.

Neurofeedback (NFB), a closed-loop brain-computer interface (BCI) technique (Dave, 2026), has emerged as a promising non-invasive intervention for ASD (Chaudhary, 2025). Unlike external modulation such as repetitive transcranial magnetic stimulation (rTMS), NFB facilitates endogenous neural plasticity by allowing individuals to self-regulate specific oscillatory activities (Sitaram et al., 2017). A randomized clinical trial conducted by Mekkawy et al. demonstrated that 40 sessions of neurofeedback training targeting the theta/beta ratio in 42 children with high-functioning autism (HFA) significantly reduced their abnormal electroencephalographic (EEG) ratios. The training concurrently improved the children’s cognitive function and three core behavioral symptoms: social interaction, thinking, and attention, with positive correlations observed between electrophysiological improvements and behavioral gains.

A study by Mehdi Rezaee et al. explicitly stated that among the 12 included studies, 83% reported positive effects of neurofeedback on cognitive function, with favorable signals observed particularly in the improvement of four core cognitive domains: attention, memory, executive function, and language ability.

Shemaila Saleem et al. administered 10 weeks of ultra-low-frequency neurofeedback training (30 sessions in total) to 35 children with autism aged 7–17 years. The results showed significant improvements in the children’s executive function, processing speed, and working memory, with the effects maintained for at least 2 months; notably, female children exhibited more pronounced enhancements in processing speed. This study confirmed that ultra-low-frequency neurofeedback is a safe and non-invasive intervention for improving cognitive function in children with autism. While previous studies have demonstrated the clinical efficacy of NFB in ameliorating ASD symptoms (Akhavan, 2018), the underlying neurophysiological mechanisms remain largely confined to the power spectrum perspective (Liu et al., 2025). However, the human brain operates as a dynamic, hierarchical system where information is processed not only through local power fluctuations but through complex, multi-layered interactions between neural populations. Relying solely on static, low-order functional connectivity (LOFC) often fails to capture the high-order organizational principles and the time-varying flexibility that are crucial for complex social-cognitive functions.

To address these limitations, a multidimensional analytical framework is required to decode the black box of neural reorganization induced by NFB. Our recent work in rTMS has highlighted the value of integrating High-Order Functional Connectivity (HOFC)—representing the correlation of correlations—and dynamic Network State Entropy (NSE) to reveal deep-seated network reconfigurations (Kang et al., 2026). Nevertheless, a major challenge in pediatric ASD research is the trade-off between spatial resolution and ecological validity. Preschool children with ASD often exhibit extreme sensitivity to high-density EEG caps (e.g., 64 or 128 channels) (Swatzyna et al., 2019), leading to low compliance and excessive motion artifacts (Yu, 2025). Consequently, there is an urgent clinical need to determine whether macro-scale network fingerprints can be robustly identified using portable, low-density EEG systems, which offer higher patient tolerance and practical feasibility for long-term intervention.

In the present study, we employed a rigorous multidimensional EEG framework to investigate brain network reorganization in preschool children with ASD following an 8-channel NFB training protocol. By covering the major cortical hubs (frontal, temporal, central, and occipital), we aimed to characterize how NFB drives the autistic brain from a disorganized state toward an optimized, small-world architecture. Specifically, we utilized HOFC to capture the similarity of spatial connectivity profiles and NSE to quantify the flexibility of neural state transitions across multiple time scales. Furthermore, we sought to establish a mechanistic link between these electrophysiological metrics and clinical improvements measured by the Autism Behavior Checklist (ABC). We hypothesized that NFB-induced clinical gains are underpinned by a systematic reorganization of high-order alpha-band networks, and that these multidimensional metrics can serve as sensitive biomarkers for monitoring therapeutic responses in early-stage ASD intervention.

## 2 Methods

### 2.1 Participant characteristics and ethical approval

A total of 40 pediatric participants with ASD (mean age 5.56 years) were enrolled in this randomized, sham-controlled trial at Ningbo Rehabilitation Hospital. Using a computer-generated randomization sequence, participants were allocated into an active NFB intervention group (*n* = 20) and a sham-control group (*n* = 20). Demographic metrics showed no significant inter-group disparities in age or sex ratio. The experimental cohort received targeted EEG-based neurofeedback training, while the control cohort was exposed to a yoked-sham condition, where non-contingent visual feedback was provided to maintain the blinding of participants and guardians.

Strict inclusion criteria ensured a homogenous sample: (i) clinical diagnosis of ASD based on DSM-5 criteria (Harm et al., 2013); (ii) right-handedness; (iii) absence of secondary neurodevelopmental comorbidities (e.g., ADHD) or seizure disorders; (iv) no prior neurosurgical interventions or severe cerebral trauma; and (v) a medication-free status for at least 4 weeks prior to baseline EEG acquisition to eliminate confounding pharmacological effects on cortical oscillatory activity. Routine behavioral therapies were maintained throughout the study to ensure ethical standards of care.

The study protocol adhered to the principles of the Declaration of Helsinki (World Medical Association, 2013) and was formally approved by the institutional review board (IRB) of Ningbo Rehabilitation Hospital (No. 2023005). Comprehensive informed consent was obtained in writing from the legal guardians of all participants after a detailed briefing on the study’s scope and safety protocols.

### 2.2 Experimental procedures and neurofeedback protocol

The neurofeedback intervention was implemented using a proprietary 8-channel EEG acquisition system (Jiangxi Jielian Medical Equipment Co., Ltd., China), featuring active electrode configurations to minimize power line interference (50 Hz) and optimize the common-mode rejection ratio (CMRR) to 120 dB. To guarantee rigorous pediatric electrical safety, the amplification module implemented a strict 3 kV galvanic isolation barrier.

The 4-week NFB intervention period consisted of a five-day weekly schedule, totaling 20 sessions. Each 20-min session was structured into five sections, with each section comprising 3 min of active training interspersed with a 1-min break to mitigate cognitive fatigue. Resting-State EEG Acquisition 1 was performed at Week 1 to establish the baseline for both groups, while Resting-State EEG Acquisition 2 was conducted immediately following the intervention at week 4.

The closed-loop NFB paradigm specifically targeted alpha oscillatory activity in the prefrontal cortex. Real-time neurofeedback training indicators were derived from channels F3 and F4 using a linked-ear reference (M1/M2), which is well-suited for targeting localized neural dynamics in early-stage developmental populations. Prior to each training block, a 1-min eyes-open resting baseline was recorded to compute the session-specific baseline metrics. During the active feedback sessions, continuous EEG streams were segmented into 2-s sliding epochs with a 1-s temporal overlap to calculate the real-time alpha-band relative power. Visual rewards (video expansion) were delivered continuously when the participant’s real-time alpha power exceeded 120% of their immediate baseline level. Conversely, a visual penalty (video contraction) occurred when the power fell below 80% of the baseline. For the yoked-sham control group, the system activated an automated pseudo-value generation algorithm. The visual feedback presented to the control cohort was modulated using these uncoupled, random pseudo-values within an identical variance profile, ensuring complete blinding of participants and clinicians.

### 2.3 Behavioral assessment and EEG data acquisition

**Figure 1:**
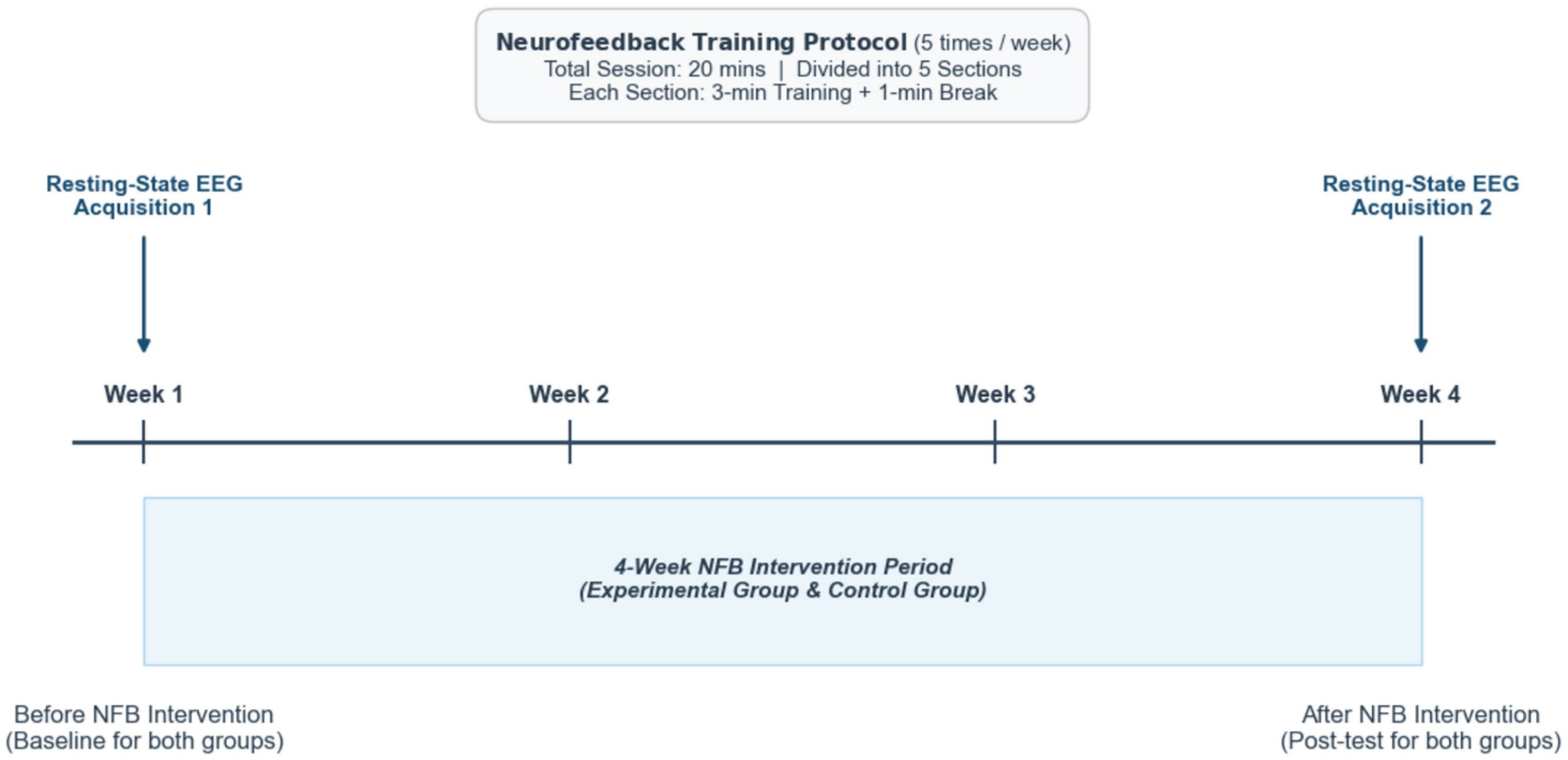
Experimental design and longitudinal timeline of the study. The schematic illustrates the 4-week NFB intervention period for both the experimental and control groups. Resting-State EEG Acquisition 1 represents the baseline assessment (Week 1), while Resting-State EEG Acquisition 2 represents the post-test evaluation (Week 4). The training protocol follows a structured format of five 20-min sessions per week, with each session divided into five sections (3-min training + 1-min break) to ensure training efficacy and participant compliance.

Prior to the neurophysiological recordings, standardized behavioral assessments were conducted under the supervision of experienced clinical professionals. Caregivers completed the Autism Behavior Checklist (ABC) to quantitatively evaluate the social impairments and behavioral symptoms of the participants (Krug et al., 1980). For both the experimental and sham-control groups, these clinical and electrophysiological data were acquired at two longitudinal time points: before the NFB intervention (baseline) and after the NFB intervention (post-intervention), as illustrated in Figure 1.

During the resting-state EEG (rs-EEG) sessions, participants were seated in a shielded, sound-attenuated, and dimly lit room. To minimize ocular and muscular artifacts, children were instructed to remain relaxed with their eyes open while maintaining a stable gaze. Approximately 5 min of continuous rs-EEG signals were recorded using a specialized 8-channel acquisition system (Resting-State EEG Acquisition 1 and 2), with electrodes positioned according to the International 10–20 system (F3, F4, T3, C3, C4, T4, O1, and O2) (Jasper, 1958). Based on the developmental characteristics of EEG in preschool children (3–6 years old) and mainstream standards in the field, this study clearly defines the alpha band as 8–13 Hz, and all real-time neurofeedback calculations and offline analyses use this frequency range without individual specific frequency offset adjustments.

The EEG signals were digitized at a sampling frequency of 1,000 Hz, utilizing the vertex (Cz) as the online reference electrode. To ensure a high signal-to-noise ratio and technical fidelity, electrode impedances were monitored in real-time and meticulously maintained below 20 kΩ throughout the recording duration.

### 2.4 Offline EEG preprocessing pipeline

Offline signal processing was performed using the EEGLAB v2024.0 toolbox within the MATLAB R2024b environment. To ensure high signal integrity despite the susceptibility of pediatric EEG to environmental and physiological artifacts, a standardized preprocessing pipeline was meticulously applied to the 8-channel data.

First, power line interference at 50 Hz was attenuated using the CleanLine algorithm. The continuous EEG signals were subsequently resampled to 250 Hz and subjected to a zero-phase Finite Impulse Response (FIR) band-pass filter with a cut-off range of 1–40 Hz. This filtering stage was designed to isolate the primary physiological frequency bands while eliminating low-frequency drifts and high-frequency instrumental noise.

To systematically address non-stationary artifacts, an automated bad-channel detection procedure was implemented. For general noise suppression, Wavelet Thresholding was employed—a method particularly suited for artifact attenuation in low-density EEG configurations. Any identified aberrant channels were subsequently recovered via spherical spline interpolation. The continuous data were then segmented into 2-s non-overlapping epochs.

To further enhance data purity, an automated epoch rejection procedure was executed based on both amplitude and statistical similarity criteria. Segments exhibiting extreme voltage fluctuations exceeding a threshold of ±150 μV were strictly excluded from further analysis. Finally, the processed data were re-referenced to the Common Average Reference (CAR) and exported for subsequent multidimensional network analysis.

### 2.5 Functional brain network architecture: static LOFC and HOFC

To investigate the neuroplastic changes induced by the intervention across distinct oscillatory regimes, functional networks were constructed for four canonical frequency bands: delta ( δ , 1–4 Hz), theta ( θ , 4–8 Hz), alpha ( α , 8–13 Hz), and beta ( β , 13–30 Hz). Phase synchronization between neural oscillations was quantified using the Phase Locking Value (PLV) to define LOFC (Watts and Strogatz, 1998).

Initially, the Hilbert Transform was applied to the preprocessed EEG time series to extract instantaneous phase dynamics for each channel (Salman et al., 2017). For each channel pair, the PLV was computed to generate an 8×8 weighted adjacency matrix, where higher coefficients denote more robust phase coupling.

The calculation formula for PLV is expressed in Equation 1:

$$\mathrm{PLV}=|\frac{1}{N}\sum_{j=0}^{N-1}e^{i(\varphi_{u}(\mathrm{j\Delta t})-\varphi_{v}(\mathrm{j\Delta t}))}|$$ (1)

Where j represented the j-th sampling point, and *N* was the total number of samples for each signal; *t* denoted the time point and ∆*t* was the sampling period. The instantaneous phases of the EEG signals *u* (*t*) and *v* (*t*) are denoted as *φ u* (*t*) and *φ v* (*t*). The PLV reflected the phase synchrony strength between paired nodes.

To further delineate the abstract organizational principles of the brain, we derived HOFC matrices, representing the correlation of connectivity profiles. Specifically, after excluding self-connections, the LOFC connectivity vectors for each node underwent a Fisher’s r-to-z transformation to ensure distributional normality. The Pearson correlation coefficient was then calculated between these transformed vectors. This hierarchical approach shifts the analytical focus from direct temporal synchrony to the topological similarity of regional connectivity patterns, capturing deeper structural regularities in the neural architecture.

### 2.6 Graph theoretical characterization

The topological properties of both LOFC and HOFC networks were characterized using graph-theoretical metrics. We prioritized four core indices to evaluate network integration and segregation (Leonardi et al., 2014): Clustering Coefficient (CC), Characteristic Path Length (CPL), Global Efficiency (GE), and Local Efficiency (LE) (Shannon, 1948). CC and LE were utilized to quantify local specialized information processing and the network’s fault tolerance within functional subgraphs. CPL and GE served as indicators of global integration, reflecting the capacity for parallel information transfer across the entire network.

The calculation formula for CC is expressed in Equation 2:

$$\mathrm{CC}=\frac{1}{n}\sum_{\mathrm{i\varepsilon \theta}}\frac{\sum_{j,\mathrm{h\varepsilon \theta}}(w_{\mathrm{ij}}^{\mathrm{pcc}}w_{\mathrm{ih}}^{\mathrm{pcc}}w_{\mathrm{jh}}^{\mathrm{pcc}})}{\sum_{\mathrm{j\varepsilon \theta}}w_{\mathrm{ij}}^{\mathrm{pcc}}(\sum_{\mathrm{j\varepsilon \theta}}w_{\mathrm{ij}}^{\mathrm{pcc}}-1)}$$ (2)

The calculation formula for CPL is expressed in Equation 3:

$$\mathrm{CPL}=\frac{1}{n}\sum_{\mathrm{i\varepsilon \theta}}\frac{\sum_{\mathrm{j\varepsilon \theta},j\neq i}d_{\mathrm{ij}}}{n-1}$$ (3)

The calculation formula for GE is expressed in Equation 4:

$$\mathrm{GE}=\frac{1}{n}\sum_{\mathrm{i\varepsilon \theta}}\frac{\sum_{\mathrm{j\varepsilon \theta},j\neq i}{\left(d_{\mathrm{ij}}\right)}^{-1}}{n-1}$$ (4)

The calculation formula for LE is expressed in Equation 5:

$$\mathrm{LE}=\frac{1}{n}\sum_{\mathrm{i\varepsilon \theta}}\frac{\sum_{\mathrm{j\varepsilon \theta},j\neq i}{\left(w_{\mathrm{ij}}^{\mathrm{pcc}}w_{\mathrm{ih}}^{\mathrm{pcc}}{\left[d_{\mathrm{jh}}(\theta_{i})\right]}^{-1}\right)}^{1/3}}{\sum_{\mathrm{j\varepsilon \theta}}w_{\mathrm{ij}}^{\mathrm{pcc}}(\sum_{\mathrm{j\varepsilon \theta}}w_{\mathrm{ij}}^{\mathrm{pcc}}-1)}$$ (5)

To preserve the full spectrum of connectivity information and mitigate the biases associated with arbitrary thresholding, all topological metrics were calculated on fully weighted networks. This ensures that subtle but significant modulations in connection strength are accurately reflected in the topological results.

### 2.7 Temporal dynamics: multi-scale sliding window analysis

To capture the time-varying reconfiguration of brain networks, we implemented a dynamic functional connectivity (dFC) framework. A multi-scale sliding window approach was employed (Bassett and Sporns, 2017), utilizing six distinct window lengths (0.5 s, 1 s, 2 s, 4 s, 6 s, and 8 s) with a 20% temporal overlap. This multi-scale strategy allows for the simultaneous detection of rapid, sub-second neural transients and slower, sustained fluctuations associated with cognitive integration. For each temporal window, dLOFC and dHOFC matrices were generated according to the procedures described in the static analysis, yielding a pseudo-continuous trajectory of the brain’s topological states.

### 2.8 Complexity of state transitions: network state entropy (NSE)

To quantify the flexibility and complexity of the brain’s dynamic transitions, we performed NSE analysis. Using time-varying participation coefficients, the K-means clustering algorithm was applied to categorize the dynamic network configurations into two fundamental topological regimes (Benjamini and Hochberg, 1995): Integration (State 1) and Segregation (State 0). To ensure the stability of these clusters, the clustering procedure was iterated 50 times.

Based on this binary state classification, we identified four possible transition patterns between consecutive windows (00, 01, 10, 11). The probability of occurrence ( $p_{i}$ ) for each transition pattern was calculated, and the Shannon Entropy formula was applied (Mentch, 2026).

$$\mathrm{NSE}=-\frac{1}{\log(M)}\sum_{i=1}^{M}p_{i}\log(p_{i})$$

Where M=4 . A higher NSE value indicates increased dynamic flexibility and a more diverse repertoire of state transitions, reflecting an optimized capacity for neural adaptation.

### 2.9 Cross-frequency interaction analysis

To evaluate the synergistic synchronization between distinct neural rhythms, we conducted a between-frequency functional connectivity analysis. Six representative cross-frequency pairs were targeted: α - β , α - δ , θ - δ , δ - β , β - θ , and α - θ . For each pair, the PLV was computed between the instantaneous phases of signals filtered in the respective frequency bands. These interactions were organized into a 16×16 supra-adjacency matrix, where off-diagonal blocks represent the strength of inter-frequency coupling. A global metric, defined as the arithmetic mean of the off-diagonal elements, was derived to provide a holistic index of cross-frequency integration.

### 2.10 Statistical framework

Statistical analyses were performed using SPSS (version 26.0). Data normality was first verified using the Kolmogorov–Smirnov (K–S) test. To evaluate the effects of the intervention, a 2×2 mixed-model Analysis of Variance (ANOVA) was implemented, with Group (Experimental vs. Control) as the between-subjects factor and Time (Baseline vs. Post-intervention) as the within-subjects factor. This model assessed significant main effects and Group × Time interactions across all multidimensional metrics. For significant interactions, post-hoc pairwise comparisons were conducted to elucidate specific treatment effects. Effect sizes were quantified using partial eta-squared ( $\eta_{p}^{2}$ ), and 95% confidence intervals (CIs) for effect sizes were reported where appropriate. To control the Type I error rate associated with multiple comparisons, all *p*-values were adjusted using the False Discovery Rate (FDR) method (Wong, 2021). Statistical significance was established at an alpha level of 0.05.

## 3 Results

### 3.1 Clinical behavioral outcomes

The therapeutic efficacy of the 4-week NFB intervention was initially evaluated using the ABC scale. As summarized in Table 1, a 2 × 2 repeated-measures ANOVA revealed significant Group × Time interaction effects for the Social Relating subscale and the ABC Total Score. Post-hoc analyses confirmed that the experimental group exhibited a substantial reduction in behavioral symptom severity following the intervention, whereas no significant changes were observed in the sham-control group (*p* > 0.05, FDR-corrected). These clinical gains provide a robust behavioral foundation for investigating the underlying neurophysiological reorganization.

**Table 1:** Statistical parameters of the Group × Time interaction effects for the Autism Behavior Checklist (ABC) subscales and total scores.

ABC scale	*F* (1,38)	*p* (FDR-corrected)	$\eta_{p}^{2}$	95% CI for $\eta_{p}^{2}$
S_1_	1.59	0.214	0.040	[0.000, 0.165]
R	5.53	0.024	0.127	[0.015, 0.312]
B	0.88	0.352	0.022	[0.000, 0.138]
L	0.18	0.675	0.005	[0.000, 0.082]
S_2_	0.17	0.684	0.004	[0.000, 0.078]
Total	4.380	0.043	0.103	[0.010, 0.284]

S1: sensory behavior, R: social relating, B: body and object, L: language and communication; S2: social and adaptive skills. *p < 0.05; rest are not significant.

### 3.2 Multidimensional reorganization of static functional networks

To elucidate the neuroplastic changes induced by NFB, we employed a hierarchical framework integrating both LOFC and HOFC functional connectivity.

#### 3.2.1 LOFC synchrony and topology

**Figure 2:**
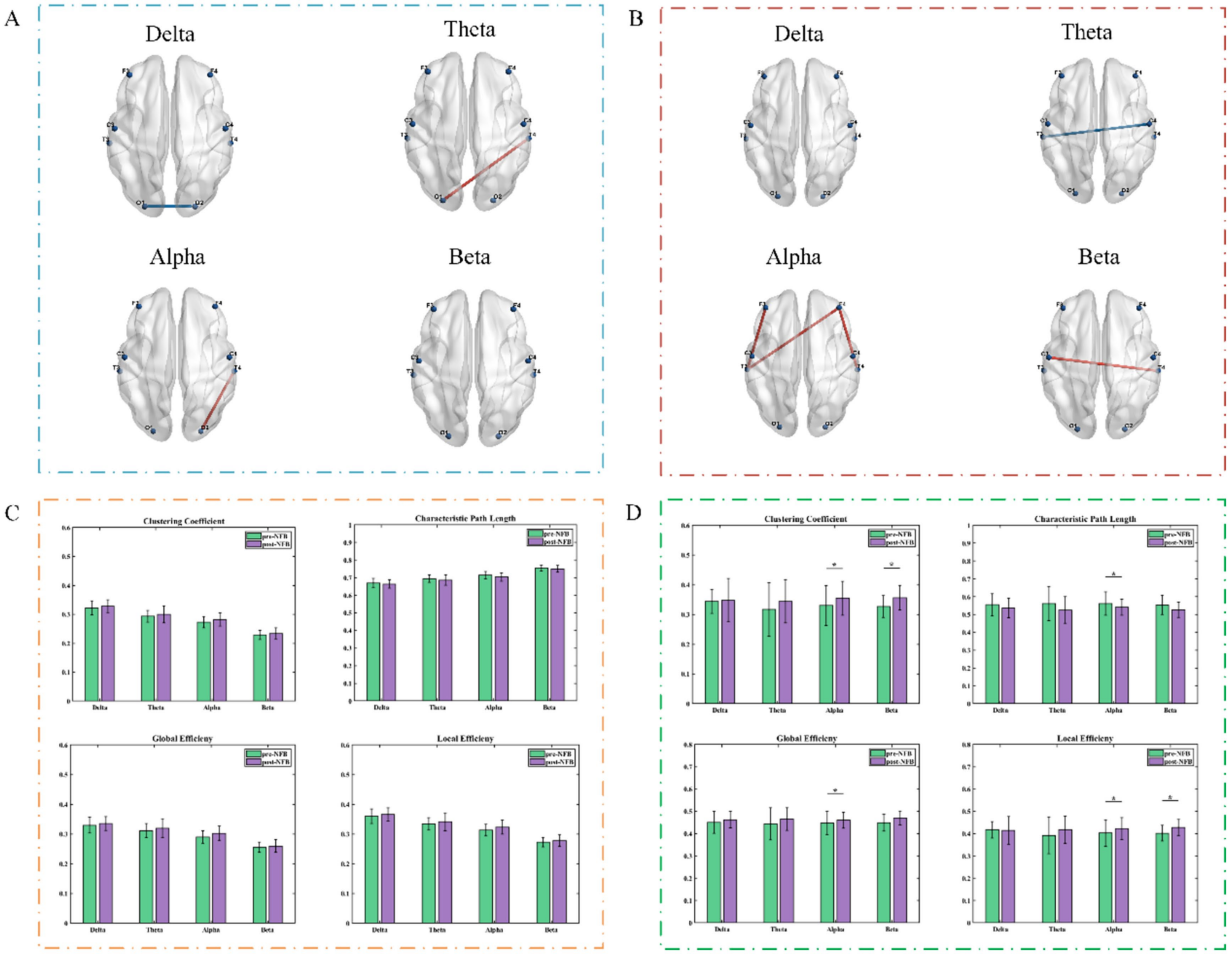
NFB-induced reorganization of static functional brain networks: A hierarchical comparison between LOFC and HOFC architectures. **(A)** Brain network maps illustrating edge-wise changes in LOFC following NFB training across the Delta, Theta, Alpha, and Beta bands. Red and blue lines represent significantly increased and decreased connectivity strengths, respectively. **(B)** Brain network maps for HOFC, showing strengthened connectivity patterns, particularly within the Alpha band. **(C)** Graph-theoretical topological properties of the LOFC network, including CC, CPL, GE, and LE. Statistical analysis revealed no significant changes across frequency bands (*p* > 0.05). **(D)** Topological alterations in the HOFC network. In the Alpha band, NFB intervention induced a significant increase in CC, GE, and LE, alongside a significant reduction in CPL (*p* < 0.05), indicating an optimized and more integrated network organization. Significant modulations were also observed in the CC and LE of the Beta band.

Edge-wise analysis of the LOFC network (representing direct phase synchronization) showed only scattered connectivity enhancements in the Alpha and Theta bands following NFB training (Figure 2A). When further characterized using graph theory, the global topological metrics—including CC, CPL, and GE/LE—did not reach statistical significance in any frequency band after multiple comparison correction (Figure 2B). These results suggest that NFB-induced changes are not fully captured by conventional low-order metrics.

#### 3.2.2 HOFC hierarchical optimization

In stark contrast, the HOFC analysis (capturing the similarity of spatial connectivity profiles) revealed a systemic and frequency-specific reorganization. As illustrated in Figure 2C, NFB intervention significantly strengthened the high-order connectivity edges within the Alpha band. Crucially, topological analysis of the Alpha-band HOFC network (Figure 2D) demonstrated a significant increase in the CC and LE, alongside a significant reduction in CPL (*p* < 0.05). This shift indicates that NFB training specifically drives the Alpha-band neural architecture toward a more efficient and integrated small-world organization.

### 3.3 Modulation of dynamic NSE

To evaluate the time-varying properties of the neural reorganization, we employed multi-scale NSE analysis.

**Figure 3:**
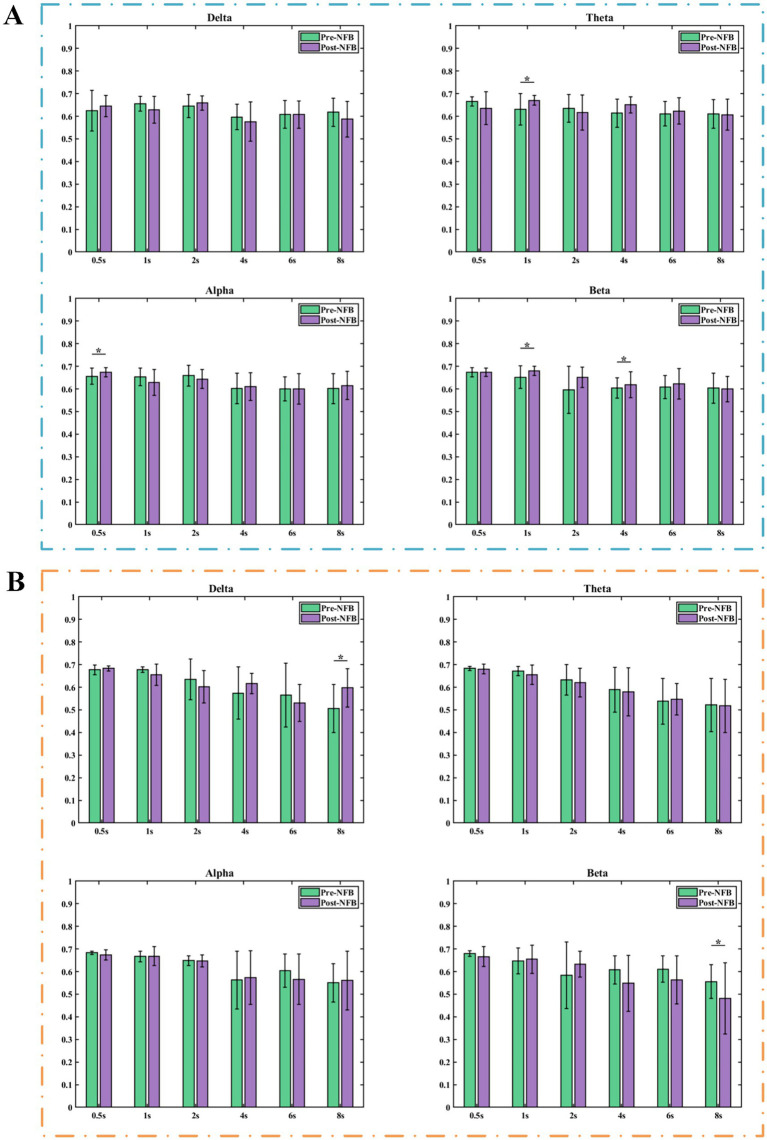
Alterations in temporal dynamics and NSE across multiple time scales. **(A)** Dynamic Network State Entropy (dNSE) of the LOFC networks calculated across six sliding-window lengths (0.5 s, 1 s, 2 s, 4 s, 6 s, and 8 s). Following NFB training, significant increases in NSE were observed in the Theta band (1 s window), Alpha band (0.5 s window), and Beta band (1 s and 4 s windows) (*p* < 0.05). **(B)** dNSE of the HOFC networks. Significant modulations were localized in the Delta and Beta bands at the longest time scale (8 s window), where NSE values exhibited a significant post-intervention increase (*p* < 0.05).

In the dynamic LOFC networks (Figure 3A), NFB training significantly modulated the state transition flexibility across multiple frequency bands (Theta, Alpha, and Beta) at sub-second and intermediate time scales (0.5 s–4 s windows). Furthermore, the dynamic high-order networks (Figure 3B) showed significant NSE alterations in the Delta and Beta bands at longer time scales (8 s window). These results suggest that NFB intervention enhances the brain’s metastability—the ability to flexibly transition between integrated and segregated states—thereby reducing the neural rigidity characteristic of ASD.

### 3.4 Synergistic cross-frequency integration

**Figure 4:**
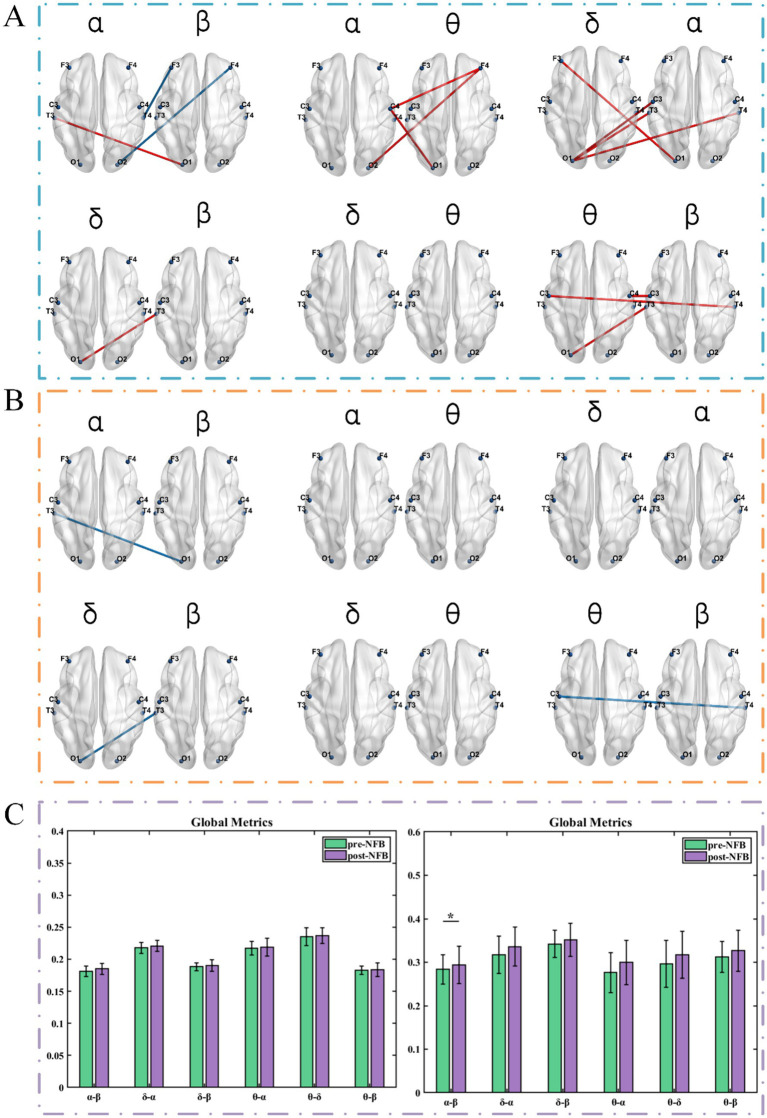
Synergistic cross-frequency interactions and global coupling metrics following NFB. **(A)** Brain network maps illustrating cross-frequency pairs with significantly increased synergistic coupling (Post-NFB > Pre-NFB, indicated by red lines). Enhancements were observed across several pairs, including α - θ and δ - α . **(B)** Brain network maps illustrating cross-frequency pairs with decreased coupling (Post-NFB < Pre-NFB, indicated by blue lines). **(C)** Quantitative analysis of global metrics for between-frequency interactions. The bar charts on the left and right represent the global coupling strength for the low-order and high-order cross-frequency networks, respectively. A statistically significant increase was observed in the high-order global metric of the α - β pair (*p* < 0.05), suggesting improved synergy between these two cognitive-related oscillatory bands. Green and purple bars represent the Pre-NFB and Post-NFB sessions, respectively. Error bars denote the standard error of the mean. The asterisk indicates statistical significance (*p* < 0.05).

Beyond within-frequency modulations, NFB intervention promoted coordination between distinct neural rhythms. Edge-wise analysis revealed a bidirectional modulation of cross-frequency interactions. As shown in Figure 4A, synergistic coupling was primarily enhanced (indicated by red lines) in the *α*-*θ* and *δ*-*α* pairs. Conversely, scattered reductions in edge-wise synchronization (indicated by blue lines) were observed in other combinations, such as the *α*-*β* pair (Figure 4B). To quantify these effects, we analyzed the global coupling metrics for both low-order and high-order between-frequency interactions (Figure 4C). While the low-order global metrics remained statistically stable, the high-order synergistic coupling strength for the *α*-*β* pair exhibited a significant increase following the NFB intervention (*p* < 0.05). This result suggests that NFB training facilitates more efficient coordination between alpha and beta oscillations. The GM values of LOFC and HOFC before and after NFB intervention are shown in the following Table 2.

**Table 2:** The GM values of LOFC and HOFC before and after NFB.

Global metrics (mean± SD)	LOFC		HOFC	
	Pre-NFB	Post-NFB	Pre-NFB	Post-NFB
*α*-*β*	0.181 ± 0.008	0.184 ± 0.009	0.283 ± 0.034	0.294 ± 0.039
*δ*-*α*	0.217 ± 0.008	0.220 ± 0.004	0.316 ± 0.043	0.336 ± 0.045
*δ*-*β*	0.188 ± 0.006	0.190 ± 0.009	0.341 ± 0.031	0.350 ± 0.038
*θ*-*α*	0.214 ± 0.010	0.218 ± 0.014	0.276 ± 0.046	0.299 ± 0.051
*θ*-*δ*	0.235 ± 0.013	0.236 ± 0.012	0.296 ± 0.054	0.317 ± 0.050
*θ*-*β*	0.182 ± 0.006	0.183 ± 0.010	0.312 ± 0.035	0.327 ± 0.048

### 3.5 Neurophysiological predictors of behavioral improvements

To establish a mechanistic link between neural reorganization and clinical outcomes, we performed Pearson correlation analyses.

**Figure 5:**
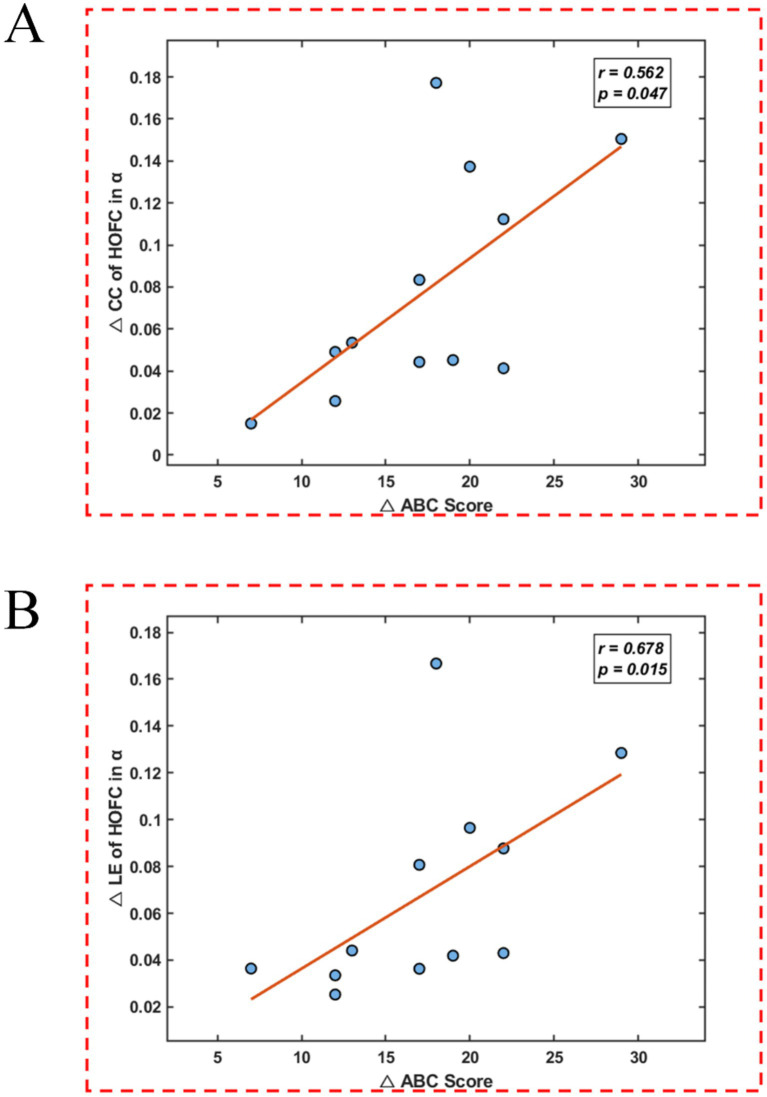
Correlations between Alpha-band high-order network reorganization and clinical symptom alleviation. Pearson correlation analyses were performed between NFB-induced topological changes and clinical gains in the active NFB group. **(A)** The post-intervention increases in the CC ( Δ CC) of the Alpha-band HOFC network was significantly positively correlated with the reduction in ABC Total Scores ( Δ ABC Score). **(B)** A robust positive correlation was observed between the increase in LE ( Δ LE) of the Alpha-band HOFC network and clinical symptom alleviation.

The most compelling finding of this study was that the optimization of Alpha-band high-order topology served as a robust predictor of therapeutic success. Specifically, the post-treatment increase in the CC of the Alpha-band HOFC network was significantly positively correlated with the reduction in ABC scores (*r* = 0.562, *p* = 0.047; Figure 5A). Moreover, the improvement in LE within the same network exhibited a strong positive correlation with clinical gains (*r* = 0.678, *p* = 0.015; Figure 5B). These results suggest that the restorative reorganization of high-order Alpha-band networks is a key neurophysiological mechanism underpinning the clinical efficacy of NFB in preschool children with ASD.

## 4 Discussion

The present study investigated the neurophysiological mechanisms underlying the clinical efficacy of alpha-band NFB in preschool children with ASD using a multidimensional EEG framework. Our findings provided robust evidence that NFB induces a localized and frequency-specific reorganization of the brain’s high-order architecture. Clinically, this reorganization was mirrored by significant improvements in social Relating and total ABC scores, which were quantitatively predicted by the optimization of alpha-band network topology.

### 4.1 Superiority of high-order hierarchy in detecting neuroplasticity

A primary finding of this study is the dissociation between LOFC and HOFC network responses. While conventional LOFC metrics failed to capture significant changes, the HOFC analysis—representing the correlation of correlations—revealed a systemic optimization of alpha-band connectivity. This discrepancy suggests that NFB-induced plasticity in the ASD brain may not manifest as a simple increase in raw phase synchrony, but rather as a refinement of the spatial connectivity patterns (Hirota and King, 2023). By strengthening the similarity of regional connectivity profiles, NFB may contribute to a topological shift that shares features with a more organized network architecture. This aligns with the under-connectivity theory of ASD (Militerni et al., 2002), suggesting that the primary deficit lies in the high-level organization of information processing hubs rather than low-level synchronization alone (Deco and Kringelbach, 2016).

### 4.2 Restoring neural flexibility and reducing rigidity

Children with ASD are often characterized by neural rigidity (Tognoli and Kelso, 2014), manifested as stereotyped behaviors and difficulty transitioning between cognitive tasks (Jensen and Mazaheri, 2010). Our dynamic NSE analysis revealed that NFB significantly increased state transition complexity, particularly in the theta, alpha, and beta bands. Higher entropy reflects a richer repertoire of functional states and enhanced metastability—the ability of the brain to flexibly engage and disengage different networks (Engel and Fries, 2010; Fornito et al., 2015). The observed increase in NSE suggests that NFB training releases the brain from a stuck or rigid state, providing a neurophysiological basis for the observed reduction in behavioral symptoms and improved cognitive adaptability.

### 4.3 Enhanced synergistic coordination between cognitive rhythms

The cross-frequency analysis highlights that NFB promotes synergistic coordination between distinct neural rhythms, specifically the α - β pair. Alpha oscillations are critical for attentional gating and top-down cognitive control (Jeste et al., 2015), while beta rhythms are associated with sensorimotor and executive processing (Hampel et al., 2023). The significant increase in high-order α - β coupling suggests that NFB facilitates a more harmonious interaction between perception and high-level cognition. This hierarchical integration is essential for complex social interactions, where the brain must simultaneously gate irrelevant sensory noise and process social cues.

### 4.4 Alpha-band HOFC as a sensitive biomarker for clinical recovery

The most compelling evidence of this study is the strong positive correlation between alpha-band HOFC metrics (CC and LE) and ABC clinical gains ( r=0.678 ). The fact that these high-order metrics exhibited a quantitative relationship with clinical symptom alleviation highlights their potential as candidate neuroimaging correlates. Unlike raw power measurements, which are prone to individual variability and noise (Kwok et al., 2022), the topological properties of the alpha-high-order network appear to capture the core neurobiological response to intervention [38]. This suggests that future precision medicine strategies for ASD could utilize macro-scale network fingerprints to monitor and adjust therapeutic protocols in real-time.

To further situate our findings within the broader clinical landscape of ASD interventions, we have provided a comparative analysis of our alpha-band NFB protocol against other state-of-the-art (SOTA) methods (Table 3). While behavioral interventions such as ABA remain the gold standard, they often require intensive time commitments and do not directly target the underlying neurobiological rigidity. Conversely, exogenous neuromodulation like rTMS offers direct brain stimulation but may face challenges regarding invasiveness and tolerability in preschool-aged children. Our study demonstrates that alpha-band NFB serves as a bridge, offering a non-invasive, endogenous regulatory mechanism that specifically drives high-order network reorganization. Importantly, the use of a portable 8-channel EEG system enhances the ecological validity of this intervention, making it highly feasible for early-stage rehabilitation in diverse clinical and home-based settings. This comparison underscores the potential of multidimensional EEG biomarkers in guiding personalized and efficient therapeutic strategies for ASD.

**Table 3:** Comparison of the current alpha-band NFB method with other state-of-the-art (SOTA) ASD interventions.

Intervention method	Primary mechanism	Targeted symptoms	Neural/biological evidence	Advantages	Limitations in pediatric ASD
Applied behavior analysis (ABA)	Behavioral reinforcement (Top-down)	Social skills, daily living, maladaptive behaviors	Indirectly through behavioral plasticity	Gold standard; extensive clinical evidence	Highly time-consuming; does not directly target neural circuits.
Pharmacotherapy	Neurotransmitter modulation (e.g., Risperidone)	Irritability, hyperactivity (Co-morbid symptoms)	Non-specific global modulation	Fast acting for secondary symptoms	Significant side effects; does not address core social deficits.
rTMS (exogenous modulation)	External electromagnetic stimulation	Social cognition, repetitive behaviors	Regulates cortical excitability/balance	Direct neuromodulation; significant efficacy	Invasive/Highintensity; poor tolerance and seizure risks in preschool children.
Traditional NFB (power-based)	Operant conditioning of EEG power	Attention, arousal, general symptoms	Changes in specific band power (e.g., Theta/Beta)	Non-invasive; endogenous regulation	Often ignores high-order network interactions and neural flexibility.
Proposed NFB (this study)	Endogenous Alpha-band Network Reorganization	Social relating and core ASD symptoms	HOFC (Small-world optimization); NSE (Increased neural flexibility)	Portable (8-ch); high compliance for preschool kids; targets core neural rigidity.	Requires multiple sessions; long-term durability needs follow-up.

### 4.5 Limitations and future directions

Despite the significant findings, several limitations should be acknowledged. First, the use of an 8-channel portable EEG system limits the spatial resolution for source localization. Low density electrode configuration may lead to systematic bias in network metrics. In the future, these findings need to be validated under higher density EEG configurations. However, our results demonstrate that macro-scale network topology is sufficient to capture robust therapeutic effects, which enhances the ecological validity and clinical translatability of this intervention for home-based or school-based settings. Second, the study focused on immediate post-treatment effects; longitudinal follow-up is necessary to determine the long-term durability of these network reorganizations.

Although we observed a significant correlation between alpha band HOFC changes and behavioral improvement, these findings are essentially exploratory and correlated. The limited sample size prevents us from making any assertions regarding predictive validity or establishing alpha band HOFC as a clinically validated prognostic biomarker. In the future, large-scale, prospective longitudinal studies must be conducted to test the generalizability and causal significance of these network level changes.

## 5 Conclusion

In conclusion, this study demonstrates that alpha-band neurofeedback effectively alleviates social impairment in children with ASD by inducing a frequency-specific reorganization of the brain’s high-order and dynamic architecture. By shifting the neural network from a rigid, disorganized state toward an optimized and flexible system, NFB offers a viable neurophysiological pathway for pediatric ASD intervention. The high correlation between high-order topological metrics and clinical gains establishes these multidimensional EEG features as promising biomarkers for monitoring therapeutic success in early-stage ASD rehabilitation.

## Data Availability

The raw data supporting the conclusions of this article will be made available by the authors, without undue reservation.
